# Identification of berberine as a direct thrombin inhibitor from traditional Chinese medicine through structural, functional and binding studies

**DOI:** 10.1038/srep44040

**Published:** 2017-03-09

**Authors:** Xing Wang, Yuxin Zhang, Ying Yang, Xia Wu, Hantian Fan, Yanjiang Qiao

**Affiliations:** 1 Beijing Key Lab of Traditional Chinese Medicine (TCM) Collateral Disease Theory Research, School of Traditional Chinese Medicine, Capital Medical University, 10 Youanmen, Xitoutiao, Beijing 100069, China; 2 Key Laboratory of TCM-Information Engineer of State Administration of TCM, School of Chinese Materia Medica, Beijing University of Chinese Medicine, 6 Central Ring South Road, Wangjing, Beijing 100102, China; 3 Core Facilities Center, Capital Medical University, 10 Youanmen, Xitoutiao, Beijing 100069, China

## Abstract

Thrombin acts as a key enzyme in the blood coagulation cascade and represents a potential drug target for the treatment of several cardiovascular diseases. The aim of this study was to identify small-molecule direct thrombin inhibitors from herbs used in traditional Chinese medicine (TCM). A pharmacophore model and molecular docking were utilized to virtually screen a library of chemicals contained in compositions of traditional Chinese herbs, and these analyses were followed by *in vitro* bioassay validation and binding studies. Berberine (BBR) was first confirmed as a thrombin inhibitor using an enzymatic assay. The BBR IC_50_ value for thrombin inhibition was 2.92 μM. Direct binding studies using surface plasmon resonance demonstrated that BBR directly interacted with thrombin with a K_D_ value of 16.39 μM. Competitive binding assay indicated that BBR could bind to the same argartroban/thrombin interaction site. A platelet aggregation assay demonstrated that BBR had the ability to inhibit thrombin-induced platelet aggregation in washed platelets samples. This study proved that BBR is a direct thrombin inhibitor that has activity in inhibiting thrombin-induced platelet aggregation. BBR may be a potential candidate for the development of safe and effective thrombin-inhibiting drugs.

Thrombin, a multifunctional serine protease generated by prothrombin cleavage, is a key enzyme in the blood coagulation cascade that can convert fibrinogen to fibrin during blood coagulation^1^. Thrombin is widely disseminated throughout the vascular system and participates in a variety of physiological and disease processes, such as blood clotting, anticoagulation, thrombosis-fibrinolysis, stroke, neurodegenerative diseases, neuroprotection, and cancer invasion and metastasis^2^,^3^,^4^,^5^. Platelet activation by thrombin is a critical factor leading to blood stasis syndrome. Thus, thrombin is a strategic target in promoting blood circulation and removing blood stasis.

Direct thrombin inhibitors, such as dabigatran, bivalirudin, argatroban, desirudin, and lepirudin, which show clinical significance in the treatment of stroke, acute venous thromboembolism, atrial fibrillation, etc., exert effects by binding directly to thrombin and are not dependent on a cofactor such as antithrombin^6^,^7^,^8^. Several kinds of direct thrombin inhibitors, such as argatroban and dabigatran etexilate, have been approved by the FDA (Food and Drug Administration) for treating cardiovascular diseases. However, they may also cause serious side effects like hemorrhage^9^. For this reason, searching new thrombin inhibitors from natural sources has been recognized as a viable and effective alternative strategy for the therapy of thromboembolic diseases^10^.

Traditional Chinese medicine (TCM) is a valuable source for drug discovery and many well-known natural products, such as artemisinin, paclitaxel, ephedrine and arsenic trioxide, separated from TCMs are playing an important role in disease treatment^11^,^12^,^13^,^14^. In this study, we describe a combination of *in silico* and *in vitro* experiments that identified a small-molecule direct thrombin inhibitor from TCM. A library of 23,033 natural compounds were screened *in silico* through pharmacophore modelling and molecular docking. The top 23 hits were evaluated for thrombin inhibition with an enzymatic assay, and berberine (BBR) showed direct thrombin inhibitory activity. Additionally, a surface plasmon resonance (SPR)-based binding study and molecular docking were carried out to characterize the interaction between BBR and thrombin. A thrombin-induced platelet aggregation assay was conducted to evaluate the *in vitro* bioactivity of BBR. The strategy used in this work provided an effective and feasible approach for identifying direct thrombin inhibitors from natural products and could promote the development of safe and effective thrombin-inhibiting drugs.

## Results

### *In silico* screening for potential thrombin inhibitors

**Figure 1:**
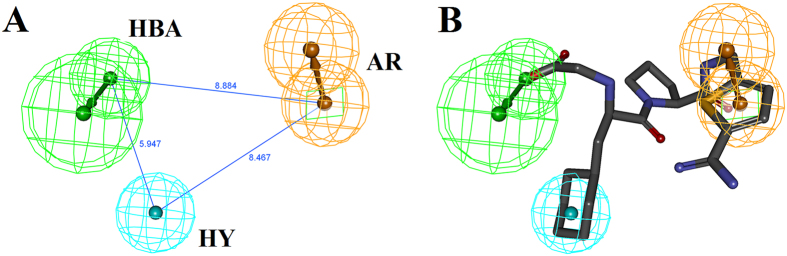
The pharmacophore model_10 of thrombin inhibitors (**A**) and the matching pattern between pharmacophore model_10 and CHEMBL377303 (**B**). The numbers in (**A**) represent the distance between the two pharmacophore features. In (**A**) and (**B**), the arrows represent the direction of the hydrogen bond groups. Grey, red, blue and yellow atoms represent carbon, oxygen, nitrogen and sulfur atoms, respectively.

Ten pharmacophore models (Table S1) were generated based on the common features of six known direct thrombin inhibitors. Model assessment studies (Table 1) indicated that Model_10 (Fig. 1A) had the highest comprehensive appraisal index (CAI) and identified effective index (N), indicating that this model had the best ability to identify active compounds and exclude inactive compounds comprehensively^15^. Model_10 contained one H-bond acceptor (HBA, marked with green), one aromatic ring (AR, marked with yellow) and one hydrophobic group (HY, marked with cyan). The best active compound (CHEMBL377303) could map all features of Model_10 with a fit value of 3.00 (Fig. 1B). Model_10 was used to screen traditional Chinese medicine database 2009 (TCMD2009, Chinese Academy of Sciences), resulting in a hit list of 93 compounds (Table S2).

**Figure 2:**
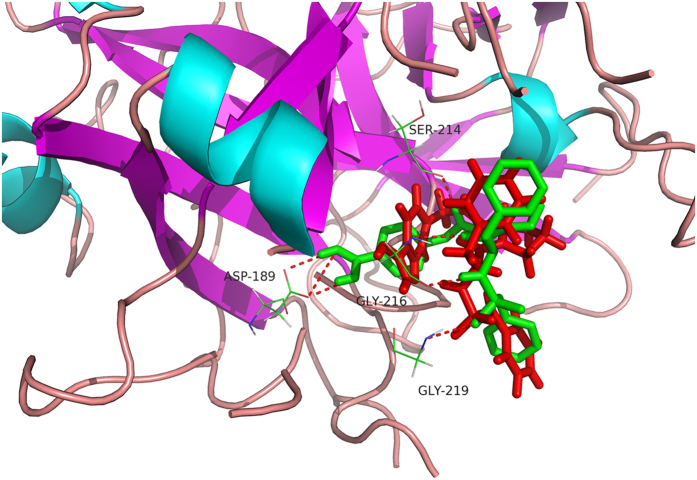
Binding conformation of ligand S49 at the active site of thrombin. The co-crystallized and re-docked conformations of ligand S49 are shown in red and green, respectively. Key residues are displayed, and hydrogen bonds are displayed as dotted lines.

**Figure 3:**
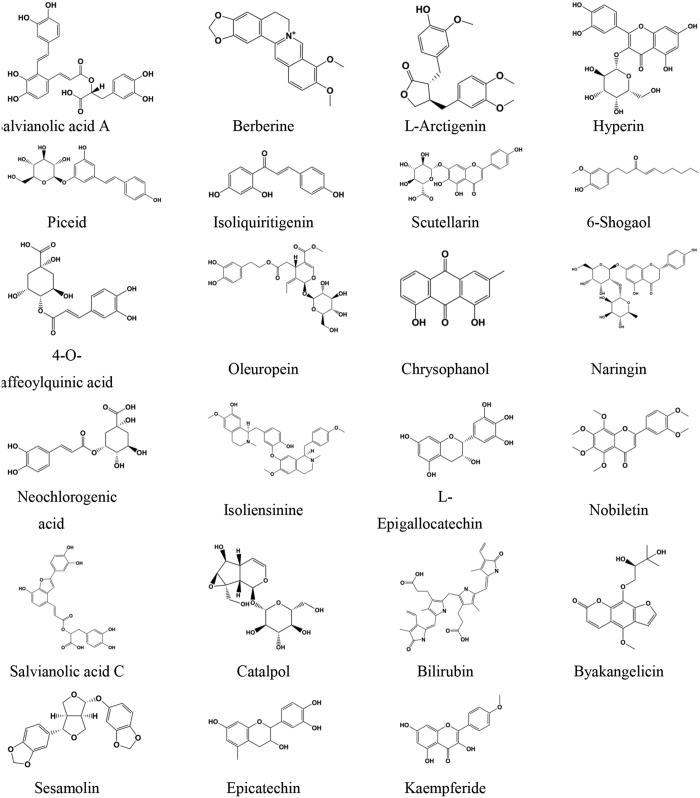
The structures of 23 potential direct thrombin inhibitor hits identified with the pharmacophore model and molecular docking.

Molecular docking was performed on the basis of the crystal structure of thrombin (PDB ID 4UFD). The known direct thrombin inhibitor ligand S49 (molecular formula: C_29_H_33_N_5_O_4_S), the structure of which was solved as a co-crystallized thrombin complex, was re-docked into the active site of thrombin to validate the reliability of the docking protocol. The result showed that S49 could bind to thrombin via H-bond interaction with Asp189, Ser214, Gly216 and Gly219 (Fig. 2), which was consistent with previous studies^16^. The low root mean-square deviation (RMSD) of the re-docked and co-crystallized conformation of S49 was calculated to be 1.48 Å (Fig. 2), which indicated that the docking protocol established in this study could reasonably predict the binding mode of a known thrombin inhibitor. Ninety-three compounds identified by the pharmacophore model were docked into the active site of thrombin, resulting in a hit list of 39 compounds with docking scores above 5.0 (Table S3). Compared with pharmacophore-based virtual screening, 23 compounds (Fig. 3) both fit the pharmacophore model well and provided reasonable docking results.

### *In vitro* screening for direct thrombin inhibitors

**Figure 4:**
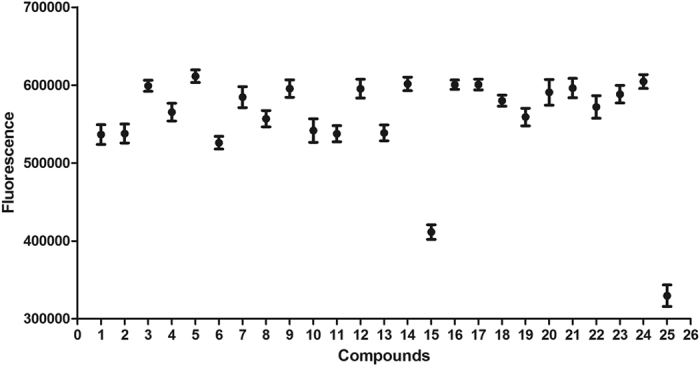
Scatterplot of the fluorescence emission values of thrombin FRET substrate solutions induced by the 23 compounds. The fluorescence emission values of thrombin FRET substrate solutions were detected in the presence of 0.13 μg/ml thrombin incubated with the 23 compounds (30 μM in the total reaction system) using the SensoLyte™ 520 Thrombin Activity Assay kit in primary screening. Compounds 1 to 23 represent the 23 test compounds, i.e., salvianolic acid A, scutellarin, hyperin, piceid, L-epigallocatechin, L-arctigenin, 4-O-caffeoylquinic acid, 6-shogaol, oleuropein, chrysophanol, naringin, kaempferide, isoliensinine, salvianolic acid C, berberine, nobiletin, isoliquiritigenin, catalpol, neochlorogenic acid, byakangelicin, sesamolin, epicatechin and bilirubin. Compounds 24 and 25 represent the negative control (transporter solution) and positive control (600 nM argatroban), respectively. All error bars indicate the SE of three replicates.

**Figure 5:**
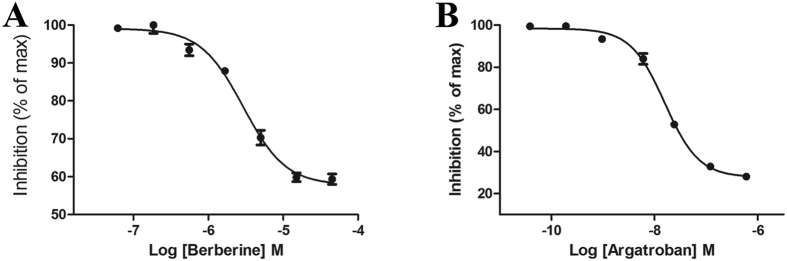
Thrombin inhibition dose-response curves for berberine (**A**) and argatroban (**B**). All error bars indicate the SE of three replicates.

The 23 compounds (30 μM FAC) were evaluated for inhibition of thrombin with an enzymatic reaction assay. The fluorescence emission values of the thrombin Förster resonance energy transfer (FRET) substrate solutions in the presence of thrombin incubated with the 23 compounds identified in the primary screen are shown in Fig. 4. Among the 23 compounds, only BBR reached 50% inhibition relative to the positive control (600 nM argatroban). The IC_50_ values of BBR and argatroban in thrombin inhibition were determined to be 2.92 μM and 15.71 nM, respectively (Fig. 5).

### SPR-based binding studies

**Figure 6:**
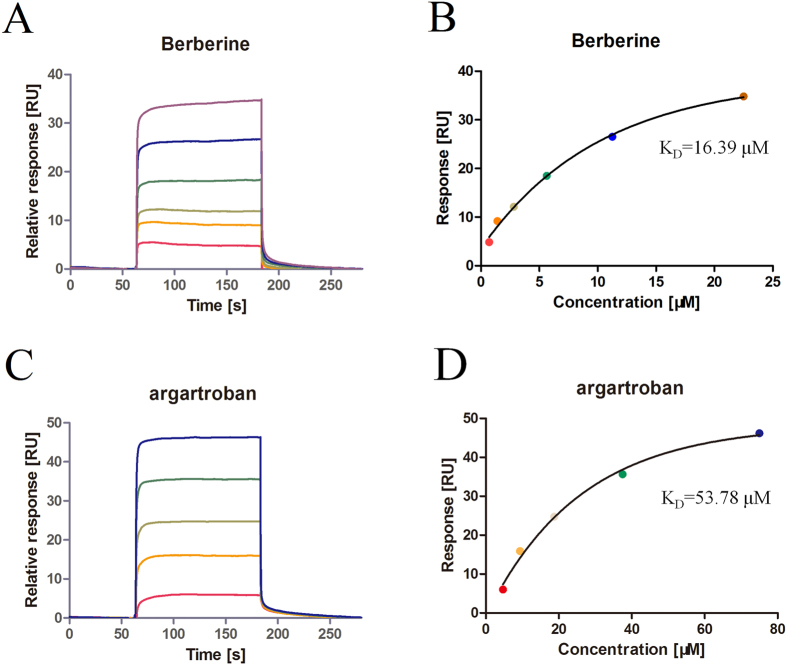
Surface plasmon resonance measurement of the binding between thrombin and BBR/argatroban. (**A**) The sensorgram of BBR binding to thrombin-immobilized chip. The BBR concentrations were 0.70, 1.40, 2.80, 5.60, 11.20 and 22.40 μM (from bottom to top). (**B**) The fitted curve for different concentrations of BBR binding to immobilized thrombin using the ‘Affinity’ model in the Biacore T200 evaluation software. (**C**) The sensorgram of argatroban binding to thrombin-immobilized chip. The argatroban concentrations were 4.70, 9.40, 18.80, 37.50 and 75.00 μM (from bottom to top). (**D**) The fitted curve for different concentrations of argatroban binding to immobilized thrombin using the ‘Affinity’ model in the Biacore T200 evaluation software.

For a deeper investigation on the interaction between BBR and thrombin, SPR-based binding analysis was performed. As shown in Fig. 6 and Table 2, BBR bound to thrombin (K_D_ = 16.39 μM) much tighter than argatroban (K_D_ = 53.78 μM) and hydroxyl alizarin (K_D_ = 1,117 μM), despite of their similar binding profile.

**Figure 7:**
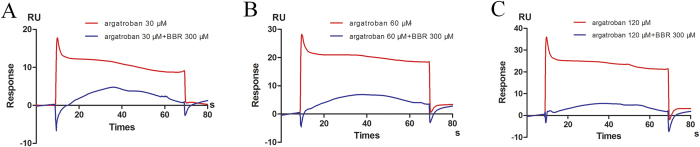
SPR competitive analysis of BBR with different concentrations of argatroban. (**A**–**C**) The sensorgram of different concentrations of argatroban binding to thrombin-immobilized chip in the presence (blue line) or absence (red line) of 300 μM BBR. (**A**) 30 μM argatroban (**B**) 60 μM argatroban (**C**) 120 μM argatroban. Results are representative of two independent experiments.

We next tested whether BBR occupies the same binding site in thrombin as argartroban. A competitive binding assay was carried out and sensorgrams from binding of mixture of argartroban and BBR were compared with those from argartroban alone. The comparison showed that the binding of argartroban to thrombin was decreased in the presence of 300 μM BBR. The inhibitory effect could be observed even with up to 120 μM argartroban (Fig. 7). Altogether, the data indicated that BBR could bind to the same argartroban/thrombin interaction site with enhanced affinity.

### Binding site of BBR in thrombin model

**Figure 8:**
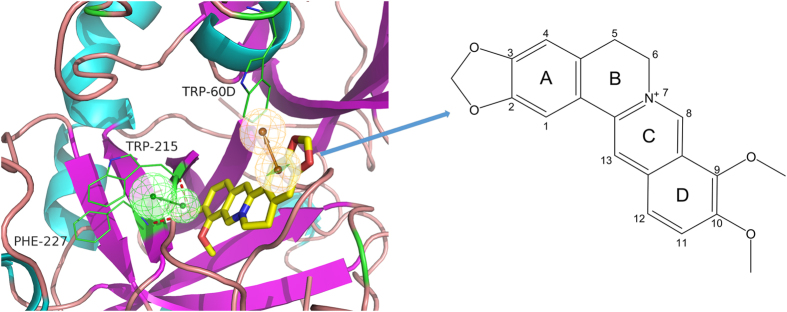
Binding modes between berberine and thrombin. Hydrogen bonding interactions are displayed as dotted lines. The green and yellow spheres represent HBA and AR, respectively.

**Figure 9:**
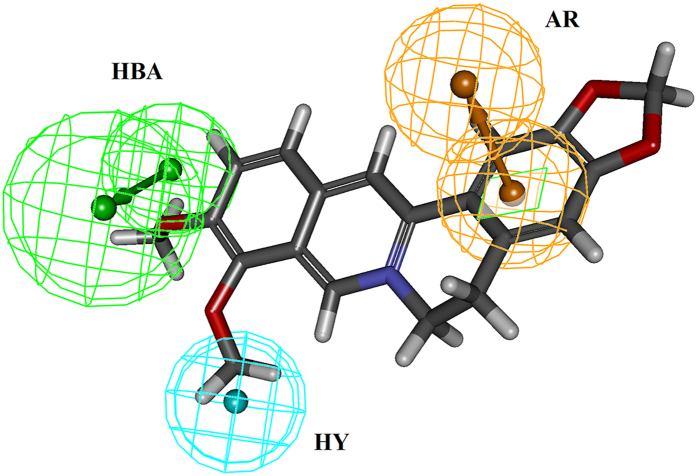
Overlay of BBR on pharmacophore model_10.

A ligand-thrombin docking model was used to reveal the mechanism underlying the molecular recognition between BBR and thrombin. The results showed that the C10 methoxy group of thrombin inserted into the catalytic centre of thrombin to form two hydrogen bond interactions with the side chain containing Phe227 and Trp215. The aromatic ring A of BBR formed π-π interactions with the side chain of Trp-60D (Fig. 8). The functional groups of BBR predicted via molecular docking were consistent with the pharmacophoric features generated with the pharmacophore model (Fig. 9).

### Antiplatelet activity of BBR

**Figure 10:**
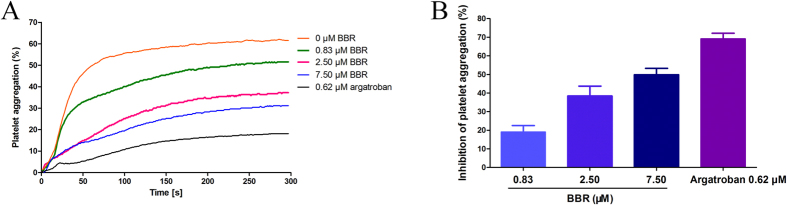
Effect of BBR on thrombin-induced platelet aggregation *in vitro*. (**A**) Thrombin-induced aggregation trace of the washed platelets preincubated with different concentrations of BBR or argatroban. (**B**) Washed platelets aggregation inhibitory percentage of different concentrations of BBR or argatroban. Washed platelets was incubated with BBR (0.83, 2.50 and 7.50 μM) or argatroban (0.62 μM) for 5 min at 37 °C. Then, 0.5 U/ml thrombin was added to trigger platelet aggregation. Data are expressed as the mean ± SD (each group, n = 3).

To evaluate the inhibitory effects of BBR on thrombin-induced platelet aggregation *in vitro*, washed platelets (WP) samples were pretreated with different concentrations of BBR. Then, 0.5 U/ml thrombin-induced platelet aggregation was monitored after the addition of test compounds for 5 minutes. As shown in Fig. 10, there were significant differences between the BBR groups and the negative control. BBR can significantly prevented platelet aggregation in WP samples.

### Cytotoxicity evaluation

**Figure 11:**
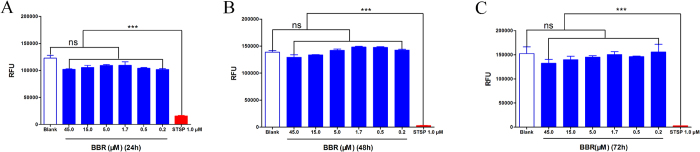
Cytotoxicity evaluation of BBR at 24 (**A**), 48 (**B**) and 72 (**C**) hours. HEK293 cells were treated with different concentrations of BBR or 1.0 μM STSP and incubated in 5% CO_2_ at 37 °C for 24 hours (**A**), 48 hours (**B**) or 72 hours (**C**). Luminescence was determined with an Envision 2100 multilabel reader to detect viability following incubation with the CellTiter-Glo reagent for 15 min. Compared to the control group, BBR showed no significant cytotoxicity on HEK293 cells after incubation for 72 hours. All error bars indicate the SE of three replicates. ns, no significant difference between the control group and the BBR groups. ***P < 0.001.

To assess the safety of BBR for its biological applicability and therapeutic uses, the cytotoxicity of BBR was evaluated with a luciferase-coupled adenosine triphosphate (ATP) quantitative assay. Staurosporine, a prototypical ATP-competitive kinase inhibitor that can induce cell apoptosis^17^, was used as a positive control in this assay. Compounds were incubated with HEK293 cells for 24, 48 or 72 hours before luminescence signal measurement. Compared to the control group, BBR showed no significant cytotoxicity on HEK293 cells after incubation for 72 hours (Fig. 11). The values represent the means and standard error of replicates from three independent experiments.

## Discussion

The combination of virtual screening, enzymatic bioassay and binding studies performed in this study showed remarkable advantages in identifying direct thrombin inhibitors from traditional Chinese herbs. Virtual screening focused on quick identification of potential active compounds from a large molecular dataset. The enzymatic bioassay focused on functional verification of direct thrombin inhibitors from the perspective of molecular biology. SPR-based binding studies focused on the physical binding kinetics of ligands and target. The platelet aggregation assay focused on *in vitro* biological efficacy. The potency of BBR was highly consistent with its ability to inhibit the thrombin target. Finally, BBR was confirmed as a direct thrombin inhibitor without cytotoxicity in HEK293 cells. Several methods were used to confirm that BBR is a direct thrombin inhibitor from multiple perspectives, providing a reference to reveal the structural and activity characteristics of a direct thrombin inhibitor.

BBR, an isoquinoline alkaloid, is a natural compound that exists in many natural herbal products, such as *Coptis chinensis, Phellodendron amurense, Chelidonium majus, Stephania cepharantha, Hydrastis canadensis,* etc. BBR is popular in TCM for its beneficial effects in human type 2 diabetes^18^, stroke^19^, hypertension^20^, inflammation^21^, microbial infection and cancer^22^, although its mechanism of action has not been studied clearly. This was the first work to identify BBR as a thrombin inhibitor, which was helpful in elucidating the mechanism underlying the anti-platelet aggregation pharmacological effect of BBR. This could be considered as a starting point for optimization to generate a dependable and efficient direct thrombin inhibitor in a follow-up study.

The essential characteristics of BBR were revealed by investigating the overlap between the pharmacophore model and docking studies. The C10 methoxy group of BBR acted as a key hydrogen bond acceptor by interacting with Phe227 and Trp215 of thrombin, which is consistent with the key amino acid residues in the active-site region of thrombin^23^. The aromatic ring A of BBR could interact with Trp-60D of thrombin through π-π interactions, suggesting that Trp-60D might be a critical amino acid residue that binds to BBR in the interaction process.

It took less than one month to carry out all the computational studies on a single computer. Together with the enzymatic bioassay, binding studies and platelet aggregation assay, the entire process took no more than two months and required significantly fewer resources than similar lead compound discovery steps in drug design programs. Therefore, this is a time- and resource-saving method for discovering direct thrombin inhibitors from natural products.

## Methods

### Pharmacophore-based virtual screening

**Figure 12:**
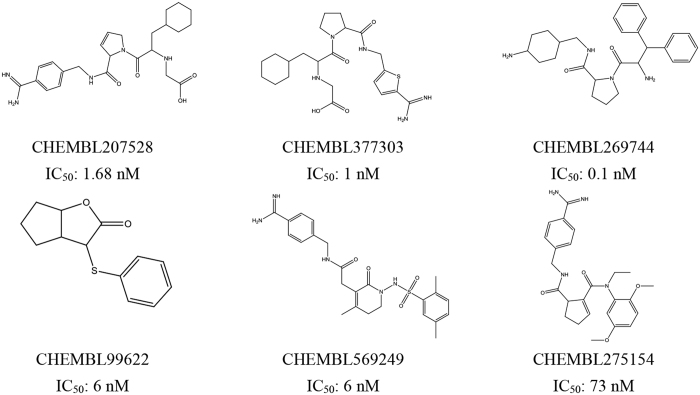
The training set used in pharmacophore model generation.

A diverse dataset of 116 experimentally known thrombin inhibitors with IC_50_ values below 1 μM were obtained from published literature^24^,^25^,^26^,^27^,^28^,^29^,^30^. All the compounds were sketched and converted to 3D structures with all proton and MMFF94 charges added using SYBYL-X 1.2. To generate the pharmacophore model, six compounds that met the following criteria were selected as a training set (Fig. 12): (a) a certain level of structural diversity; (b) high antagonistic activity in each series (with IC_50_ values below 80 nM); and (c) similar pharmacophore features to ensure a similar binding mechanism. The other 110 antagonists were utilized as a validation set.

The Common Feature Pharmacophore Generation protocol in Discovery Studio v3.5 (Accelrys, San Diego, CA, USA) was used to generate the ligand-based pharmacophore models. A principal value of 2 and a maximum omit feature value of 0 was assigned to the six compounds in the training set. Energy minimization was performed using the CHARMM force field for all the compounds. Poling algorithm was used to generate a maximum of 255 diverse conformations with the threshold of 20 kcal·mol^−1^ above the calculated lowest energy for each compound in the training set. Conformers were generated using the diverse conformer generation protocol running with the best conformer generation option as available in DS. All the training set compounds associated with their conformations were used to generate the pharmacophore using the common feature pharmacophore generation module in DS. The feature mapping protocol was used to identify common features shared by the training set. As predictors, hydrogen-bond acceptor (HBA), hydrogen-bond donor (HBD), hydrophobic (HY) and aromatic ring (AR) features were selected during pharmacophore generation. Ten possible pharmacophore models having one more arrangement of constituent features were generated in each pharmacophore running. The pharmacophore models were classified according to the ranking scores. Redundant hypotheses having the exact same chemical characteristics and nearly equal distances between these functions were deleted^31^.

### Pharmacophore validation and virtual screening

The pharmacophore models were validated with an external decoy set database consisting of 100 experimentally known thrombin inhibitors and 256 inactive compounds retrieved from the literature, utilizing the built-in parameters from our previous studies^32^. Four parameters (*i.e.* A%, Y%, N and CAI) were calculated to evaluate the generated models according to the following formula^15^.


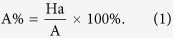



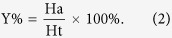



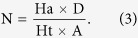



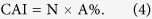


D is the number of compounds in the external database. A is the number of active compounds. Ht is the number of hits and Ha is the number of active hits. A% represents the ability to identify active compounds from the external database. Y% represents the proportion of active hits in total hits. N represents the ability to distinguish active compounds from non-active compounds. CAI was utilized to evaluate the models comprehensively. The model with the highest CAI was used to screen TCMD2009.

A total of 23,033 compounds from TCMD 2009 were extracted and converted into 3D conformers using the CONCORD module in SYBYL-X 1.2 software (Tripos, Inc., St. Louis, MO, USA). The structures of all 23,033 compounds were energy minimized using the Tripos force field (Powell method and 0.05 kcal/mol Å energy gradient convergence criteria) and assigned an electrostatic charge with the Gasteiger-Hückel method. All the energy-minimized structures were stored as a 3D database in TCMD2009.

Virtual screening was performed using the search 3D database module in DS, with the minimum interference distance set to 1 Å and the search method set to best. All other protocol parameters were kept as the default settings. Fit value was calculated to indicate the matching degree of each ligand on the pharmacophoric features. A higher fit value suggests a better alignment between ligand’s conformer and pharmacophore model.

### Molecular docking-based virtual screening

Surflex-Dock, a well-recognized method in the field of molecular docking^33^,^34^, was used to perform virtual screening and calculate the ligand-receptor interaction. The X-ray crystal structure of thrombin (PDB code: 4UFD. Resolution: 1.43 Å) resolved recently was designated to be the docking template^35^. Crystallographic water molecules in the structure were deleted and hydrogen atoms were added. Using the standard parameters employed in SYBYL-X 1.2 software package, the structure was energy minimized using AMBER7 F99 force field.

To check the accuracy of the docking program, ligand S49 was flexibly re-docked into the active site of thrombin. The RMSD of the re-docked and co-crystallized conformation of ligand S49 was calculated according to the following formula:


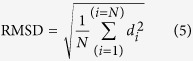


where d is the distance between N pairs of equivalent atoms excluding hydrogens. A lower RMSD indicates a higher overlap between the re-docked and co-crystallized conformation of ligand S49.

The active site of thrombin was defined as the protomol generated using the steric hydrophobic group (CH_4_), the hydrogen bond group (C =O), and the hydrogen acceptor (N-H) within 0.5 Å of the ligand S49 binding site. After validation, the compounds identified by pharmacophore model were docked into the active site of thrombin using the default parameters in SYBYL-X 1.2 software. After each docking run, the best ten docked conformers were sorted in a molecular spreadsheet. They represent binding affinities in −log 10(Kd) based on surflex-dock scoring function (crash score (also pKd units), polar score, D-score, PMF-score, G-score, ChemSco and CScore)^36^. The common hits through ligand- and structure-based virtual screening were further evaluated using thrombin inhibition assay.

### Thrombin inhibition assay

To evaluate the thrombin-inhibitory activity of the hits, a fluorescence assay utilizing a SensoLyte 520 Thrombin Activity Assay Kit (AnaSpec, Inc., San Jose, CA) was performed following the manufacturer’s instructions. This kit contained a novel 5-FAM/QXL 520 (fluorophore/quencher pair) FRET modified substrate (approximately 6 amino acids) that can be cleaved by thrombin at the Arg-Gly (R-G) site. The FRET substrate can be cleaved into two separate fragments, resulting in the release of 5-FAM fluorescence, which can be monitored at an excitation/emission = 490 nm/520 nm^37^. Argatroban (CAS No. 74863-84-6), a known direct thrombin inhibitor^38^, was used as a reference positive control for thrombin activity assay. All the test compounds were acquired from the National Institutes for Food and Drug Control (China) for thrombin inhibition assay. The purity of all the compounds was over 98% on the basis of HPLC analysis.

Ten microlitres of compound solution (300 μM in assay buffer) and 40 μL thrombin solution (0.33 μg/ml in assay buffer) were sequentially added to each well of a black, flat- bottom, non-binding 96-well plate (Corning #3650, Corning, NY). The plate was incubated for 10 min at 37 °C. Fifty microlitres of thrombin-substrate solution (6.67 μM in assay buffer) was then added to each well. The reaction mixture was incubated for 1 hour at 37 °C. After incubation, the fluorescence (excitation 490 nm, emission 520 nm) of each mixture was detected with a 2104 EnVision Multilabel Reader (PerkinElmer).

For enzymatic reaction, the thrombin solution treated with vehicle buffer alone was used as negative control. The thrombin solution incubated with argatroban (60 μM in assay buffer) was used as positive control. Furthermore, 100 μL hits solutions (300 μM in assay buffer) were served as test compound control to avoid false results due to compound autofluorescence. All fluorescence readings are taken into account in relative fluorescence units (RFU). The experimental data were analyzed by GraphPad Prism software (version 5, Graphpad Software Incorporation, USA).

### Binding affinity measurement using SPR

SPR binding studies were performed using a Biacore T200 biosensor system (GE Healthcare, Uppsala, Sweden). All the SPR-based materials were acquired from GE Healthcare. Thrombin from human plasma (T6884), dimethyl sulfoxide (DMSO, W387520) were purchased from Sigma-Aldrich Chemical Co. (St. Louis, MO, USA).

Thrombin was diluted in 10 mM sodium acetate buffer at pH 5.0 and immobilized on a CM5 chip using an amine coupling kit. The immobilization of thrombin on a CM5 chip was performed according to the BIA applications handbook^39^. A total of 8419 RU of immobilized proteins was obtained. Sensor preparation and interaction analyses were performed at 25 °C in a PBS-P [10 mM phosphate buffer, 137 mM NaCl, 2.7 mM KCl, 0.05% P20 (pH 7.4)] running buffer containing 5% DMSO.

Tested compounds were prepared in a two-fold dilution concentration series by running buffer containing 5% DMSO (0.35–11.25 μM for berberin, 4.7–75 μM for argatroban and 1.17–37.50 μM for hydroxy alizarin). Argatroban and hydroxy alizarin were set as positive control and negative control, respectively. Solvent-correction procedures were included to compensate for any DMSO-related bulk refractive index variations. Reference flow cell without immobilized thrombin served as a non-specific binding control. Biacore traces were baseline subtracted and the signal was presented in sensorgrams and measured in RU. Empirically in the BIAcore technology, 1 ng of analyte bound at the surface gives a response of 1000RU^2^,^40^. Equilibrium constants (K_D_) were calculated using the ‘affinity’ model in Biacore T200 evaluation software version 2.0.

Using the same thrombin-immobilized CM5 chip, SPR competitive binding assays were performed following “ABA” method in a Biacore S200 biosensor system (GE Healthcare). Argartroban (30, 60 or 120 μM) were injected with or without BBR (300 μM) over the surface of the chip for 60 seconds at 30 μL/min. For test of mixture binding, PBS-P containing BBR (300 μM) was used as running buffer instead of PBS-P alone. The response units were measured before the end of injection.

### Inhibition of thrombin-induced platelet aggregation assay

To further evaluate the anti-thrombin activities of BBR, inhibition of thrombin-induced platelet aggregation was measured using the turbidimetric method^41^. Male rabbits (weight 2.88 kg) were anesthetized with 10% chloral hydrate and blood was collected from the aortic neck and anticoagulated with sodium citrate (109 mM; 9:1, v/v). PRP was obtained by centrifugation of collected blood at 190 g for 10 min. The platelets were isolated from PRP by centrifugation for 10 min at 650 g, resuspended and washed twice in Ca^2+^-free Tyrode’s buffer (138 mM NaCl, 2.68 mM KCI, 11.9 mM NaHCO_3_, 0.36 mM NaH_2_PO_4_, 0.49 mM MgSO_4_, 5.5 mM glucose, pH7.5)^42^. Washed platelets were then resuspended in Tyrode’s buffer including 1.8 mM CaCl_2_ at a density of 2 × 10^7^ platelets/ml. The trial protocol was approved by the Ethical Committee of Capital Medical University, China.

In brief, 300 μL WP was preincubated with BBR (final concentrations of 0.83, 2.50 and 7.50 μM), argatroban (final concentrations of 0.62 μM) or normal saline (control group) for 5 min at 37 °C. Aggregation was initiated by the addition of 10 μL of thrombin (final concentrations of 0.5 U/ml). Aggregation was recorded at 37 °C for 5 min using a four-channel platelet aggregation analyser (LBY-NJ4, Beijing Precil Instrument Co., Ltd., China). Platelet aggregation inhibitory percentage was calculated using the following equation: Inhibitory percentage (%) = (1 − A_t_/A_0_) × 100%. A_t_ is the platelet aggregation percentage of the test sample, and A_0_ is the platelet aggregation percentage of the control sample.

### Luciferase-coupled ATP quantitative assay

HEK293 cells were seeded at 5.0 × 10^3^ per well into 96-well clear-bottom black plates and incubated in 5% CO_2_ at 37 °C overnight. Different concentrations of BBR or 1.0 μM STSP were added to the 96-well plates and incubated with the cells in 5% CO_2_ at 37 °C for 24 hours, 48 hours or 72 hours. Luminescence was determined with an Envision 2100 multilabel reader to detect viability following incubation with CellTiter-Glo reagent for 15 min.

### Statistics

For platelet aggregation and cytotoxicity evaluation experiment, statistical analyses were performed by one-way analysis of variance (ANOVA) followed by Dunnett’s multiple comparison test. A value of p < 0.05 was considered as statistically significant.

## Additional Information

**How to cite this article:** Wang, X. *et al*. Identification of berberine as a direct thrombin inhibitor from traditional Chinese medicine through structural, functional and binding studies. *Sci. Rep.* **7**, 44040; doi: 10.1038/srep44040 (2017).

**Publisher's note:** Springer Nature remains neutral with regard to jurisdictional claims in published maps and institutional affiliations.

## Supplementary Material

Supplementary Dataset

## Figures and Tables

**Table 1:** Assessment results for each pharmacophore model.

Model	Ht^a^	Ha^b^	A (%)^c^	Y (%)^d^	N^e^	CAI^f^
Model_01	173	90	90.00	52.02	1.85	1.67
Model_02	161	90	90.00	55.90	1.99	1.79
Model_03	163	89	89.00	54.60	1.94	1.73
Model_04	166	91	91.00	54.82	1.95	1.78
Model_05	167	90	90.00	53.89	1.92	1.73
Model_06	169	91	91.00	53.85	1.92	1.74
Model_07	180	91	91.00	50.56	1.80	1.64
Model_08	172	91	91.00	52.91	1.88	1.71
Model_09	168	91	91.00	54.17	1.93	1.75
**Model_10**	**155**	**92**	**92.00**	**59.35**	**2.11**	**1.94**

^a^Ht is the number of hits.

^b^Ha is the number of active hits.

^c^A% represents the ability to identify active compounds from the external database (A% = Ha/A, while A is the number of active compounds in the external database).

^d^Y% represents the proportion of active hits in total hits (Y% = Ha/Ht).

^e^N represents the ability to distinguish active compounds from non-active compounds (N = (Ha/Ht)/(A/D), while D is the number of compounds in the external database).

^f^CAI was utilized to evaluate the models comprehensively (CAI = N × A%).

**Table 2:** The binding parameters of the interaction between immobilized thrombin and test compounds.

Compounds	CAS No.	K_D_ (M)
BBR	2086-83-1	16.39
Argatroban	74863-84-6	53.78
Hydroxyalizarin	81-54-9	>200
Gallic acid	149-91-7	>200
Panaxatriol	32791-84-7	>200
