# Supplementary material for: Identification of berberine as a direct thrombin inhibitor from traditional Chinese medicine through structural, functional and binding studies
Source: Sci Rep. 2017 Mar 9;7:44040. doi: 10.1038/srep44040 (PMC5343495; doi:10.1038/srep44040)
Supplement: Supplementary Dataset [file srep44040-s1.doc]

**Supplementary materials: Identification of berberine as a** **direct thrombin inhibitor from traditional Chinese medicine through structural, functional and binding studies**

**Xing Wang^1#^, Yuxin Zhang^2#^, Ying Yang^3^, Xia Wu^1^, Hantian Fan^1^ & Yanjiang Qiao^2^**

^1^ Beijing Key Lab of Traditional Chinese Medicine (TCM) Collateral Disease Theory Research, School of Traditional Chinese Medicine, Capital Medical University, 10 Youanmen, Xitoutiao, Beijing 100069, China;

^2^ Key Laboratory of TCM-Information Engineer of State Administration of TCM, School of Chinese Materia Medica, Beijing University of Chinese Medicine, 6 Central Ring South Road, Wangjing, Beijing 100102, China;

^3^ Core Facilities Center, Capital Medical University, 10 Youanmen, Xitoutiao, Beijing 100069, China.

^#^ These authors contributed equally to this work.

Correspondence and requests for materials should be addressed to X.Wang (email: [kingstar1016@sina.com](mailto:kingstar1016@sina.com)) or Y.J.Q. (email: [yjqiao@bucm.edu.cn](mailto:yjqiao@bucm.edu.cn))

**Table S1.** Generation of the pharmacophore models of thrombin inhibitors.

| **Model** | **Features ^a^** | **Rank score ^b^** | **Direct Hit** | **Partial Hit** | **Max Fit ^c^** |
| --- | --- | --- | --- | --- | --- |
| 01 | RHAA | 55.018 | 111111 | 0 | 3 |
| 02 | RHA | 48.068 | 111111 | 0 | 3 |
| 03 | RHA | 48.068 | 111111 | 0 | 3 |
| 04 | RHA | 47.946 | 111111 | 0 | 3 |
| 05 | RHA | 47.309 | 111111 | 0 | 3 |
| 06 | RHA | 46.964 | 111111 | 0 | 3 |
| 07 | RHA | 46.182 | 111111 | 0 | 3 |
| 08 | RHA | 45.971 | 111111 | 0 | 3 |
| 09 | RHA | 45.817 | 111111 | 0 | 3 |
| 10 | RHA | 45.602 | 111111 | 0 | 3 |

^a^ H represents the hydrophobic group; A represents the hydrogen bond acceptor; ^b^ A higher rank score means a better-quality pharmacophore model; ^c^ Max Fit means the number of pharmacodynamic characteristics that match the molecules.

**Table S2.** The hits through pharmacophore model_10-based virtual screening.

| **TCMD ID** | **Compounds** | **Source** | **Fit value** |
| --- | --- | --- | --- |
| 2008 | Aurantioobtusin | Cassia tora | 2.87 |
| 19201 | Salvianolic acid A | Salvia miltiorrhiza | 2.82 |
| 5414 | 3,4-Di-O-caffeoylquinic acid | Gardenia jasminoides | 2.81 |
| 18739 | Rhamnose | Panax ginseng | 2.8 |
| 2892 | Caffeine | Camellia sinensis | 2.79 |
| 19587 | Scutellarin | Scutellaria baicalensis | 2.78 |
| 8099 | (-)-Gallocatechin | Musa acuminata | 2.78 |
| 17981 | Protopanaxadiol | Panax ginseng | 2.75 |
| 10887 | Hyperin | Uncaria rhynchophylla | 2.73 |
| 17283 | Piceid | Polygonum multiflorum | 2.72 |
| 6921 | L-Epigallocatechin | Camellia sinensis | 2.72 |
| 2918 | 4-O-Caffeoylquinic acid | Helianthus annuus | 2.71 |
| 1621 | L-Arctigenin | Arctium lappa | 2.71 |
| 19846 | 6-Shogaol | Zingiber officinale | 2.7 |
| 11648 | Isorhamnetin | Ginkgo biloba | 2.68 |
| 1928 | Astilbin | Smilax glabra | 2.68 |
| 16080 | Oleuropein | Ligustrum lucidum | 2.68 |
| 12925 | Lithospermic acid | Lithospermum officinale | 2.67 |
| 4630 | Danshensu | Salvia miltiorrhiza | 2.67 |
| 19497 | Schizandrin C | Schisandra chinensis | 2.67 |
| 2303 | Berberine | Coptis chinensis | 2.65 |
| 4400 | Curdione | Curcuma aromatica | 2.64 |
| 8095 | Gallic acid | Paeonia albiflora | 2.64 |
| 8282 | Genistein 7-glucoside | Pueraria lobata | 2.62 |
| 753 | (R)-(-)-Agrimol B | Agrimonia pilosa | 2.62 |
| 3615 | Chrysophanol | Rheum officinale | 2.61 |
| 18180 | Puerarin | Pueraria lobata | 2.6 |
| 7768 | Ferulic acid | Ligusticum chuanxiong | 2.58 |
| 20100 | Sophoridine | Sophora flavescens | 2.57 |
| 15286 | Naringin | Poncirus trifoliata | 2.56 |
| 17145 | Phillyrin | Forsythia suspensa. | 2.55 |
| 11491 | Isoliensinine | Nelumbo nucifera | 2.55 |
| 12015 | Kaempferide | Kaempferia galanga | 2.55 |
| 9071 | Guanosine | Pinellia ternata | 2.54 |
| 3774 | Citrulline | Saussurea lappa | 2.54 |
| 19750 | Sennoside B | Cassia angustifolia | 2.53 |
| 19203 | Salvianolic acid C | Salvia miltiorrhiza | 2.52 |
| 15635 | Nobiletin | Citrus reticulata | 2.5 |
| 3696 | Cinnamic alcohol | Narcissus tazetta | 2.42 |
| 11504 | Isoliquiritigenin | Glycyrrhiza uralensis | 2.4 |
| 19110 | Safflomin A | Carthamus tinctorius | 2.4 |
| 1476 | Apigenin | Ginkgo biloba | 2.36 |
| 3306 | Catalpol | Rehmannia glutinosa | 2.36 |
| 12847 | Limonin | Coptis chinensis | 2.36 |
| 15363 | Neochlorogenic acid | Plantago major | 2.32 |
| 8906 | Gomisin A | Schisandra chinensis | 2.3 |
| 11505 | Isoliquiritin | Glycyrrhiza uralensis | 2.26 |
| 18222 | Purpurin | Rubia cordifolia | 2.18 |
| 20685 | Tanshinone I | Salvia miltiorrhiza | 2.16 |
| 2833 | Byakangelicin | Angelica dahurica | 2.14 |
| 19780 | Sesamolin | Sesamum indicum | 2.13 |
| 6854 | Epicatechin | Ginkgo biloba | 2.09 |
| 2102 | Baicalein | Scutellaria baicalensis | 2.08 |
| 20444 | Succinic acid | Stemona tuberosa | 2.07 |
| 18411 | Quercitrin | Ginkgo biloba | 2.06 |
| 2374 | Bilirubin | Bos taurus domesticus | 2.03 |
| 15265 | Narcissin | Narcissus tazetta | 1.99 |
| 8908 | Gomisin C | Schisandra chinensis | 1.98 |
| 12020 | Kaempferol | Ginkgo biloba | 1.97 |
| 12908 | Liquiritigenin | Glycyrrhiza uralensis | 1.94 |
| 19749 | Sennoside A | Cassia angustifolia | 1.93 |
| 16776 | Peimisine | Fritillaria siechuanica | 1.93 |
| 6923 | Epigallocatechin 3-gallate | Camellia sinensis | 1.89 |
| 1618 | Arbutin | Aristolochia debilis | 1.86 |
| 2678 | Brucine | Strychnos nux-vomica | 1.79 |
| 3911 | Colchicine | Colchicum autumnale | 1.77 |
| 12798 | Liensinine | Nelumbo nucifera | 1.76 |
| 5045 | Demethoxycurcumin | Curcuma longa | 1.75 |
| 19777 | Sesamin | Sesamum indicum | 1.74 |
| 9455 | Hesperetic acid | Salvia miltiorrhiza | 1.65 |
| 2439 | Bisdemethoxycurcumin | Curcuma longa | 1.61 |
| 18759 | Rhein | Rheum officinale | 1.6 |
| 11680 | Isorhyncophylline | Uncaria rhynchophylla | 1.57 |
| 22144 | Tussilagone | Tussilago farfara | 1.57 |
| 8817 | Glycine | Codonopsis pilosula | 1.56 |
| 4606 | Daidzin | Glycine max | 1.54 |
| 1623 | Arctiin | Arctium lappa | 1.44 |
| 19081 | Rutaecarpine | Evodia rutaecarpa | 1.38 |
| 1048 | γ-Aminobutyric acid | Bufo bufo gargarizans | 1.3 |
| 2376 | Bilobalide A | Ginkgo biloba | 1.23 |
| 8289 | Genkwanin | Glycyrrhiza glabra | 1.23 |
| 17762 | Praeruptorin C | Peucedanum praeruptorum | 1.22 |
| 6776 | Emodin | Rheum officinale | 1.09 |
| 16532 | Paeonol | Paeonia moutan | 1.05 |
| 7852 | Folic acid | Ligusticum chuanxiong | 0.86 |
| 11462 | Isoimperatorin | Angelica dahurica | 0.85 |
| 5088 | Demethylnobiletin | Citrus reticulata | 0.84 |
| 16264 | Ostruthin | Peucedanum ostruthium. | 0.59 |
| 6815 | Ephedrine | Ephedra sinica | 0.37 |
| 5178 | Deoxygomisin A | Schisandra chinensis | 0.3 |
| 1969 | Atractylodin | Atractylodes lancea | 0.26 |
| 3402 | Cephalomannine | Taxus baccata | 0.22 |
| 967 | Aloeemodin | Rheum officinale | 0.14 |

**Table S3.** The hits through docking-based virtual screening.

| **TCMD ID** | **Compounds** | **Source** | **Total Score** | **Crash^a^** | **Polar^b^** |
| --- | --- | --- | --- | --- | --- |
| 2374 | Bilirubin | Bos taurus domesticus | 8.51 | -2.75 | 1.04 |
| 19780 | Sesamolin | Sesamum indicum | 7.33 | -2.28 | 2.09 |
| 19201 | Salvianolic acid A | Salvia miltiorrhiza | 6.98 | -2.53 | 3.96 |
| 19846 | 6-Shogaol | Zingiber officinale | 6.94 | -1.22 | 1.5 |
| 16080 | Oleuropein | Ligustrum lucidum | 6.87 | -3.13 | 4.96 |
| 12798 | Liensinine | Nelumbo nucifera | 6.84 | -3.06 | 1.58 |
| 6854 | Epicatechin | Ginkgo biloba | 6.56 | -1.63 | 2.97 |
| 1621 | L-Arctigenin | Arctium lappa | 6.34 | -1.98 | 1.81 |
| 2303 | Berberine | Coptis chinensis | 6.27 | -2.1 | 0.2 |
| 15286 | Naringin | Poncirus trifoliata | 6.27 | -4.22 | 4.62 |
| 15363 | Neochlorogenic acid | Plantago major | 6.18 | -1.95 | 5.42 |
| 16264 | Ostruthin | Peucedanum ostruthium. | 6.18 | -1.3 | 1.07 |
| 11504 | Isoliquiritigenin | Glycyrrhiza uralensis | 6.16 | -0.98 | 2.36 |
| 2439 | Bisdemethoxycurcumin | Curcuma longa | 5.95 | -0.64 | 0.66 |
| 2918 | 4-O-Caffeoylquinic acid | Helianthus annuus | 5.91 | -1.91 | 3.2 |
| 6923 | Epigallocatechin 3-gallate | Camellia sinensis | 5.89 | -3.06 | 5.2 |
| 5178 | Deoxygomisin A | Schisandra chinensis | 5.88 | -1.44 | 0 |
| 5045 | Demethoxycurcumin | Curcuma longa | 5.85 | -0.81 | 1.33 |
| 19203 | Salvianolic acid C | Salvia miltiorrhiza | 5.73 | -0.85 | 2.44 |
| 10887 | Hyperin | Uncaria rhynchophylla | 5.63 | -2.62 | 3.86 |
| 18759 | Rhein | Rheum officinale | 5.59 | -1.23 | 2 |
| 7852 | Folic acid | Ligusticum chuanxiong | 5.53 | -1.25 | 4.48 |
| 17283 | Piceid | Polygonum multiflorum | 5.42 | -1.32 | 3.64 |
| 1048 | γ-Aminobutyric acid | Bufo bufo gargarizans | 5.42 | -0.22 | 2.59 |
| 22144 | Tussilagone | Tussilago farfara | 5.41 | -1.29 | 0 |
| 19081 | Rutaecarpine | Evodia rutaecarpa | 5.39 | -0.65 | 2.71 |
| 6776 | Emodin | Rheum officinale | 5.38 | -0.56 | 1.76 |
| 6921 | L-Epigallocatechin | Camellia sinensis | 5.38 | -0.79 | 1.73 |
| 2833 | Byakangelicin | Angelica dahurica | 5.36 | -0.87 | 2.39 |
| 15265 | Narcissin | Narcissus tazetta | 5.26 | -1.52 | 2.37 |
| 12015 | Kaempferide | Kaempferia galanga | 5.13 | -0.57 | 0.99 |
| 11491 | Isoliensinine | Nelumbo nucifera | 5.12 | -3.6 | 0.01 |
| 3615 | Chrysophanol | Rheum officinale | 5.12 | -1.11 | 0.89 |
| 4606 | Daidzin | Pueraria lobata | 5.12 | -2.8 | 3.44 |
| 967 | Aloeemodin | Rheum officinale | 5.1 | -1.52 | 0.9 |
| 3306 | Catalpol | Rehmannia glutinosa | 5.08 | -1.28 | 3.86 |
| 17762 | Praeruptorin C | Peucedanum praeruptorum | 5.04 | -1.14 | 0 |
| 15635 | Nobiletin | Citrus reticulata | 5.04 | -1.47 | 0.05 |
| 19587 | Scutellarin | Scutellaria baicalensis | 5.01 | -1.87 | 1.05 |

^a^ Crash means the degree of inappropriate penetration by the ligand into the protein and of interpenetration (self-clash) between ligand atoms that are separated by rotatable bonds. Crash scores close to 0 are favorable. Negative numbers indicate penetration; ^b^ Polar means the contribution of the polar interactions to the total score. The polar score is useful for excluding docking results that make no hydrogen bonds.
